# Pelvic floor biomechanical reconstruction for moderate to severe pelvic organ prolapse: two-year outcomes of anatomical restoration and urinary continence

**DOI:** 10.3389/fsurg.2026.1828203

**Published:** 2026-07-09

**Authors:** Jihong Shen, Xunguo Yang, Zhenhua Gao, Ling Li, Daoming Tian, Yuan Li, Jiangna Gu, Hongcheng Li, Qian Luo, Xingqi Wang

**Affiliations:** 1 Department of Urology, The First Affiliated Hospital of Kunming Medical University, Kunming, China; 2 Southern Hospital of Southern Medical University, Guangzhou, China

**Keywords:** biomechanical reconstruction, occult stress urinary incontinence, pelvic organ prolapse, surgical technique, ultrasound

## Abstract

**Objective:**

To evaluate the anatomical restoration, changes in urinary control function, and safety of pelvic floor biomechanical reconstruction in patients with moderate to severe pelvic organ prolapse (POP).

**Materials and methods:**

This retrospective study analyzed the clinical data of 135 patients with POP-Q stage ≥ III who underwent pelvic floor biomechanical reconstruction between January 2022 and December 2023. Based on preoperative symptoms and urodynamics, patients were classified as occult stress urinary incontinence (OSUI, *n* = 32), no SUI with negative urodynamics (*n* = 41), and clinically confirmed SUI (*n* = 62). Follow-up assessments were conducted at 3 months, 1 year, and 2 years postoperatively. Outcomes included POP-Q indicators (Aa, Ba, C, D, Ap, Bp, Gh, Pb, TVL), the Pelvic Floor Distress Inventory-20 (PFDI-20), and the Patient Global Impression of Improvement (PGI-I). Transperineal three-dimensional ultrasound was performed preoperatively and at 3 months postoperatively to measure the urethral rotation angle (URA), retrovesical angle (RVA), bladder neck descent (BND), and levator hiatus area (LHA). Perioperative and follow-up complications, POP recurrence, and SUI outcomes were also recorded.

**Results:**

All procedures were completed successfully. All POP-Q indicators improved significantly at 3 months, 1 year, and 2 years compared with baseline (all *P* < 0.001). At 3 months, ultrasound parameters decreased significantly (URA 81.5 ± 25.9° to 38.0 ± 13.7°; RVA 164.7 ± 19.5° to 106.9 ± 18.9°; BND 37.1 ± 13.3 to 12.7 ± 6.6 mm; LHA 30.7 ± 5.2 to 17.2 ± 4.8 cm^2^; all *P* < 0.05). PFDI-20 scores decreased from 144.6 ± 44.5 preoperatively to 52.1 ± 21.3 (3 months), 13.7 ± 11.1 (1 year), and 8.1 ± 8.5 (2 years) (*P* < 0.001). At 2 years, 94.8% of patients reported “very much improved” or “much improved”. Overall postoperative SUI symptom rate was 3.7%. POP recurrence occurred in 2.2%. Major adverse events were uncommon: intraoperative complications 3.0% and mesh exposure 1.5%.

**Conclusion:**

Pelvic floor biomechanical reconstruction provides durable anatomical correction with favorable continence outcomes and low recurrence, with manageable morbidity.

## 1 Introduction

Pelvic organ prolapse (POP) and stress urinary incontinence (SUI) frequently coexist, sharing common etiological foundations (1). Studies indicate that nearly 70% of patients with severe POP experience varying degrees of SUI symptoms (2). Most POP cases primarily manifest as anterior vaginal wall prolapse or cystocele. Such anatomical changes lead to relaxation of the supporting structures of the bladder neck and posterior bladder wall, weakening the support for the mid-to-distal urethra. This subsequently causes functional shortening of the urethral length and ultimately results in SUI (3). However, in some POP patients, the prolapsed organs cause mechanical obstruction of the urethra. SUI symptoms only become apparent after the prolapse is reduced. This phenomenon is defined as occult stress urinary incontinence (OSUI) (4). Additionally, SUI may also emerge following POP surgical treatment, termed *de novo*SUI (5, 6).

Current mainstream surgical procedures for moderate to severe POP, such as sacrocolpopexy and transvaginal mesh (TVM), primarily focus on restoring apical support and static anatomical position in their design philosophy. They often provide insufficient reconstruction of the overall pelvic floor mechanical balance and urinary continence mechanisms (7–10). Consequently, the incidence of postoperative *de novo*SUI or the unmasking of OSUI remains relatively high, with literature reporting rates of 24%–33% (11–13). To mitigate postoperative incontinence risk, a concomitant midurethral sling (MUS) procedure is often performed during POP repair in clinical practice (14). Although this combined strategy can reduce SUI occurrence to some extent, it also increases the risks of complications such as voiding dysfunction, sling erosion, and bladder injury (15, 16). Therefore, a significant challenge in contemporary pelvic floor reconstructive surgery is how to effectively repair the prolapse while simultaneously restoring the patient's intrinsic physiological urinary continence function, rather than over-relying on synthetic slings.

The pelvic floor biomechanical reconstruction surgery employed in this study emphasizes the holistic reconstruction of the anterior and posterior compartments. It systematically repairs the levator ani complex and perineal body, aiming to reshape the stress fulcrum of the bladder neck and the functional support of the urethra. The goal is to improve urinary control function alongside anatomical restoration. This retrospective study analyzed 135 patients with moderate to severe POP who underwent this procedure. It systematically evaluated anatomical outcomes, urinary control-related urodynamic parameters, subjective symptom improvement, and safety. The aim is to explore the feasibility and safety of this approach as a comprehensive treatment strategy for moderate to severe POP.

## 2 Materials and methods

### 2.1 Study design and participants

This single-center retrospective study adhered to the principles of the Declaration of Helsinki and was approved by the Ethics Committee of The First Affiliated Hospital of Kunming Medical University [IRB No. (2024) Ethical Review L No. 240]. We retrospectively analyzed the clinical data of POP patients who underwent pelvic floor biomechanical reconstruction between January 2022 and December 2023. Inclusion criteria were: ① Female patients with POP-Q stage ≥ III; ② Patients who underwent pelvic floor biomechanical reconstruction; ③ Availability of complete preoperative urodynamic data; ④ Availability of complete preoperative and postoperative follow-up data. ⑤Patients who provided written informed consent for their clinical data to be used for research purposes. Exclusion criteria included: a history of pelvic radiotherapy, long-term use of immunosuppressive drugs, severe psychiatric or psychological disorders, and loss to follow-up during the study period. A total of 135 patients were ultimately included.

### 2.2 Surgical procedure

Pelvic floor biomechanical reconstruction comprised two main steps: anterior compartment mesh implantation and posterior compartment reconstruction (combined levator ani and perineal body repair). All procedures in this cohort were performed by a single, highly experienced senior surgeon. A video of the surgical procedure is available as supplementary material (Supplementary Video 1).

Step 1

**Figure 1:**
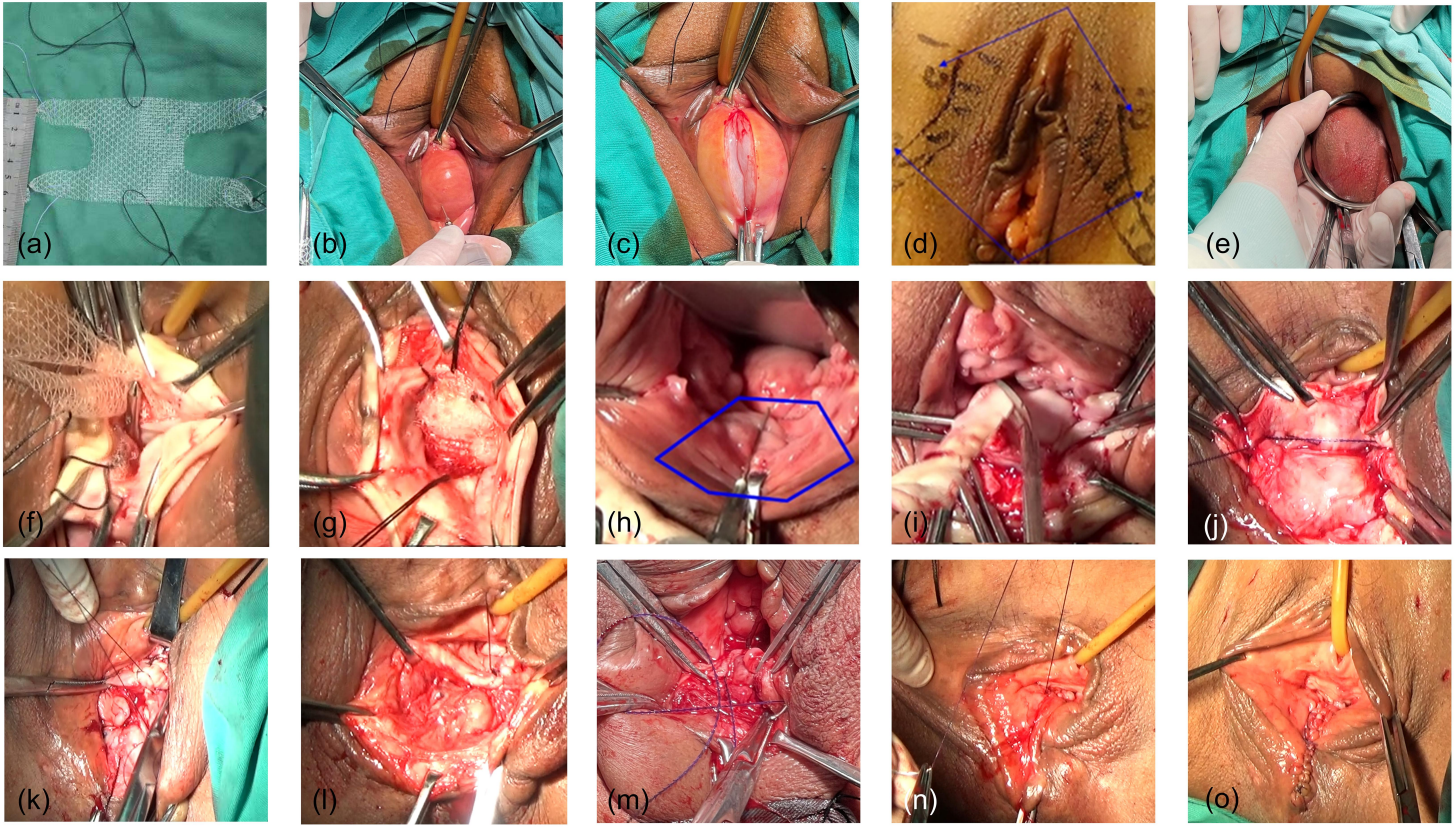
Surgical procedures for pelvic floor biomechanical reconstruction. **(a)** A polypropylene mesh was cut to obtain four long arms (approximately 2 cm × 5 cm) and a central rectangular part (approximately 5–8 cm × 5 cm). **(b)** Water separation. **(c)** Make an incision on the anterior vaginal wall. **(d)** Anatomical landmark. **(e,f)** The mesh was placed by retracting the introducer needles. **(g)** Fix the mesh on the vaginal wall to prevent the mesh from shifting. **(h)** Make a hexagonal incision on the posterior vaginal wall. **(i)** The excess vaginal epithelium was trimmed. **(j)** Reconstruct the levator ani muscle and the genital hiatus. **(k)** The upper half of the posterior vaginal epithelium was closed. **(l,m)** Reconstruct the perineal body. **(n)** The lower half of the posterior vaginal epithelium was closed. **(o)** The mesh was tightened and cut.

**Figure 2:**
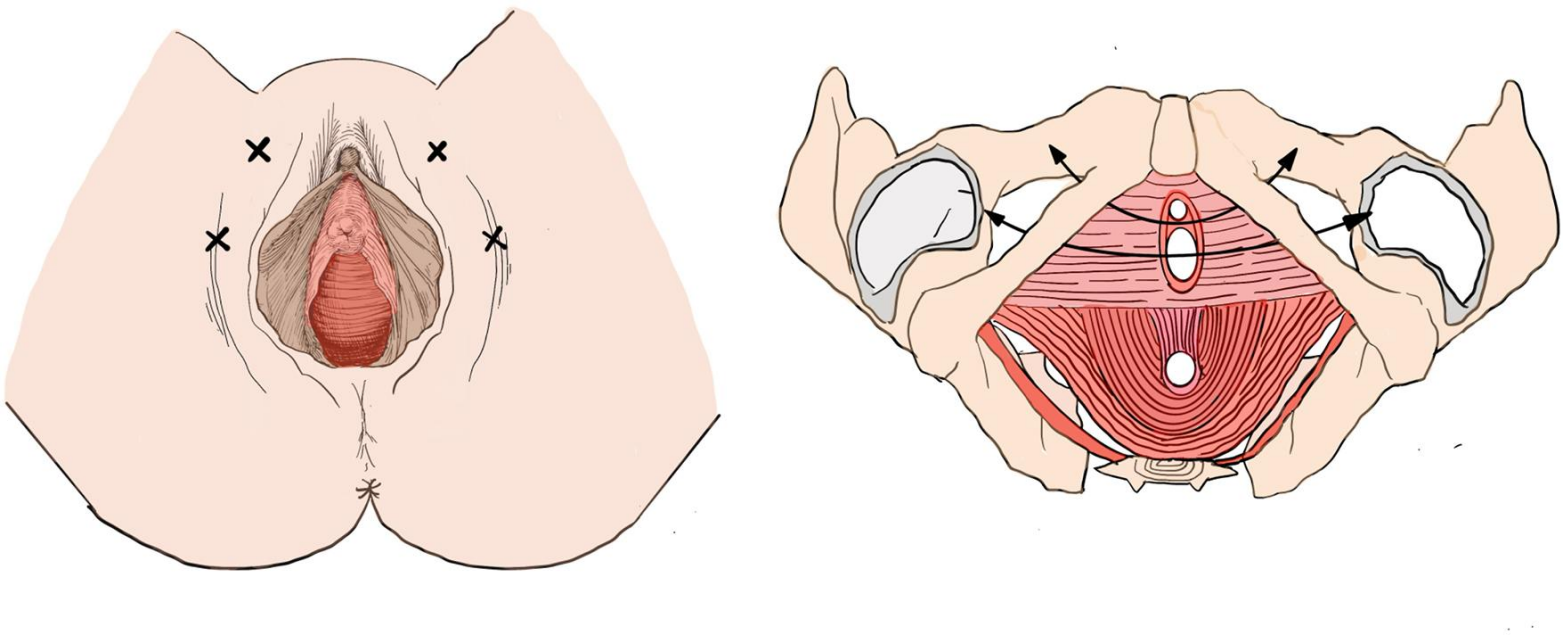
Anatomical landmark. The descending pubic ramus serves as the primary anatomical landmark. The upper puncture point is located 0.5 cm above the most superior aspect of the pubic symphysis, and the lower puncture point is located 0.5 cm below the ischiopubic ramus.

A polypropylene mesh (Pelvimesh®; Herniamesh®, Chivasso, Italy) was trimmed into four long arms (approximately 2 × 5 cm each) and a central rectangular portion (approximately 5–8 × 5 cm) (Figure 1a). Adrenaline-containing saline was injected submucosally along the anterior vaginal wall to adequately separate the plane between the vaginal wall and the bladder (Figure 1b). A longitudinal incision was made on the anterior vaginal wall, extending from the suburethral groove to approximately 2 cm anterior to the cervix (Figure 1c). The anterior vaginal space was dissected posteriorly to the descending pubic ramus. The descending pubic ramus served as the anatomical landmark. The upper puncture point was identified as 0.5 cm above the most superior aspect of the pubic symphysis. The lower puncture point was identified as 0.5 cm below the ischiopubic ramus (Figures 1d, 2). Guided by the index finger, the trocar was passed through the obturator membrane from the obturator foramen on each side, and the four arms of the mesh were placed (Figures 1e,f). During the surgical procedure, the color of the urine were closely monitored to evaluate potential bladder injury, and routine cystoscopy was not performed. The four corners of the mesh were fixed with 2-0 absorbable sutures, ensuring it lay flat from the mid-urethra to 2 cm anterior to the cervix (Figure 1g). The vaginal mucosa was then folded and closed over the mesh using a 3-0 barbed suture, completely covering the mesh.

Step 2

The vaginal caliber was maintained at approximately 3 cm in diameter. For elderly women without sexual activity, the vaginal caliber could be appropriately reduced. A hexagonal incision was designed on the posterior vaginal wall, with the widest part of the flap corresponding to the levator ani plane (Figure 1h). The incision was made just above the hymenal ring, and the rectovaginal space was dissected up to the level of the rectouterine pouch (pouch of Douglas) (Figure 1i). The levator plate and levator ani muscles were approximated horizontally below the cervix using a 2-0 barbed suture to reduce the levator hiatus area (Figure 1j). The vaginal mucosa was sutured to the levator ani plane using 2-0 absorbable sutures (Figure 1k). Reconstruction of the perineal body and the external anal sphincter was continued using a 2-0 barbed suture (Figures 1l,m). The vaginal incision was closed with 2-0 absorbable sutures (Figure 1n). Finally, the four arms of the anterior mesh were tightened to achieve a natural, tension-free state (Figure 1o).

### 2.3 Case and follow-up data collection

#### 2.3.1 Preoperative data

The following preoperative data were collected: ① Basic demographic and clinical information, including age, body mass index (BMI), menopausal status, obstetric history, sexual activity, vaginal delivery history, previous surgical history, and comorbidities; ② POP staging: Patients were staged using the Pelvic Organ Prolapse Quantification (POP-Q) system (17). This involved recording the nine specific measurements: Aa, Ba, C, D, Ap, Bp, Gh, Pb, and TVL; ③ Based on preoperative clinical evaluation and urodynamic results, patients were categorized into three groups: Group A: 32 patients with no clinical SUI symptoms but with urodynamically confirmed OSUI. Group B: 41 patients with neither clinical SUI symptoms nor urodynamic evidence of SUI. Group C: 62 patients with clinically confirmed SUI preoperatively; ④ The transperineal ultrasound was performed using a GE Voluson E10 system equipped with a RAB 4–8 MHz volume transducer. The parameters were measured and calculated at both the resting state and during the Valsalva maneuver. These parameters included Urethral Rotation Angle (URA), Retrovesical Angle (RVA), Bladder Neck Descent (BND), and Levator Hiatus Area (LHA), considered dynamic indicators for assessing urinary continence-related structures (18–20); ⑤ Quality of life (QoL) was assessed using the Pelvic Floor Distress Inventory-20 (PFDI-20) questionnaire. Higher scores indicate more severe pelvic floor dysfunction and a greater impact on QoL.

#### 2.3.2 Postoperative data

Patients were followed up at 3 months, 1 year, and 2 years postoperatively. ① Anatomical restoration: The POP-Q system was used to evaluate anatomical outcomes. Recurrence was defined as a POP-Q stage ≥ II postoperatively, regardless of whether clinical symptoms were present. ② At the 3-month follow-up, patients underwent repeat 3D pelvic floor ultrasound to measure the four parameters: URA, RVA, BND, and LHA. ③ QoL improvement: The PFDI-20 questionnaire was administered again to assess changes in QoL. ④ Patient Global Impression of Improvement (PGI-I): Patients rated their perceived improvement on a scale with five levels: “Very much improved”, “Much improved”, “Minimally improved”, “No change”, and “Worse”. Patients reporting “Very much improved” or “Much improved” were considered subjectively satisfied. ⑤ All adverse events during the perioperative period and follow-up were recorded. These included bladder or rectal injury, vaginal wall injury, postoperative hemorrhage, urinary retention, urgency urinary incontinence, thigh/inguinal pain, and mesh exposure. Mesh-related complications were classified according to the International Urogynecological Association/International Continence Society (IUGA/ICS) joint complication classification system (16).

### 2.4 Statistical analysis

Statistical analysis was performed using SPSS 27.0 software (IBM Corp., Armonk, NY, USA). Continuous variables were tested for normality with the Shapiro–Wilk test. Normally distributed data are presented as mean ± standard deviation, while non-normally distributed data are presented as median (interquartile range). Categorical variables are expressed as number (n) and percentage (%). Comparisons of POP-Q indicators and PFDI-20 scores between preoperative and postoperative time points (3 months, 1 year, 2 years) were performed using repeated-measures analysis of variance (ANOVA). If the sphericity assumption was violated, the Greenhouse-Geisser correction was applied. *post-hoc* pairwise comparisons used the Bonferroni correction. Comparisons of ultrasound parameters between preoperative and 3-month postoperative measurements were performed using paired t-tests. A two-sided *P*-value of <0.05 was considered statistically significant.

## 3 Results

### 3.1 Patient baseline characteristics

The demographic and surgery-related characteristics of the patients are summarized in Table 1. Among the 135 patients, 86 (63.7%) had POP-Q stage III and 49 (36.3%) had POP-Q stage IV.

**Table 1:** Baseline characteristics of study participants.

Characteristic	All patients (*n* = 135)
Age(years), mean ± SD(range)	66.7 ± 11.1 (44–86)
BMI(kg/m^2^), mean ± SD(range)	23.2 ± 2.8 (17.5–30.9)
Postmenopausal, n(%)	95 (70.4%)
Parity, mean ± SD(range)	4.6 ± 2.0 (1–7)
Sexual activity, n(%)	49 (36.3%)
Prior vaginal deliveries, n(%)	135 (100.0%)
Prior hysterectomy, n(%)	6 (4.4%)
Prior POP surgery, n(%)	2 (1.5%)
Prior SUI surgery, n(%)	0 (0%)
Chronic obstructive pulmonary disease, n(%)	18 (13.3%)
Hypertension, n(%)	58 (43.0%)
Diabetes mellitus, n(%)	24 (17.8%)
Pelvic organ prolapse–quantification stage, *n* (%)	
III	86 (63.7%)
IV	49 (36.3%)
Urinary dysfunction, n(%)	
Clinical incontinence	62 (45.9%)
Clinical incontinence	73 (54.1%)
OSUI	32 (23.7%)
No urodynamic SUI	41 (30.4%)

Data are mean ± SD (range); n(%). SD, Standard Deviation; BMI, Body Mass Index; POP, Pelvic Organ Prolapse; OSUI, Occult Stress Urinary Incontinence; SUI, Stress Urinary Incontinence.

### 3.2 Perioperative complications

All surgeries were completed successfully without major hemorrhage or infection. The average operative time was 74.8 ± 8.8 min (60–90 min). The average estimated blood loss was 28.5 ± 11.6 mL (10–60 mL), and the average hospital stay was 5.3 ± 1.0 days (4–7 days). Four patients (3.0%) experienced intraoperative complications: one case (0.7%) of bladder perforation, which was managed by re-puncture after withdrawing the trocar, followed by 7 days of indwelling urinary catheterization; two cases (1.5%) of vaginal sidewall perforation, managed by re-puncture and repair; and one case (0.7%) of rectal injury, managed by povidone-iodine irrigation, layered closure of the fistula, placement of a rectal tube, followed by 5–7 days of fasting, parenteral nutrition, and antibiotic therapy. All affected patients recovered well without further issues. The urinary catheter was routinely removed on postoperative day 3. Four patients (3.0%) developed acute urinary retention, which resolved after another 3 days of catheterization, likely related to postoperative local edema. The summary of perioperative complications is shown in Table 2.

**Table 2:** Perioperative and follow-up complications.

Complications	All patients *n* = 135	3-month n＝135	1-year n＝135	2-year n＝135
Perioperative complications				
Bladder injury, n(%)	1 (0.7%)			
Vaginal injury, n(%)	2 (1.5%)			
Rectal injury, n(%)	1 (0.7%)			
Severe bleeding, n(%)	0 (0%)			
Acute urinary retention, n(%)	4 (3.0%)			
Follow-up complications				
Thigh or groin mild pain		5 (3.7%)	2 (1.5%)	1 (0.7%)
Overactive bladder		7 (5.2%)	3 (2.2%)	1 (0.7%)
Urgent urinary incontinence		6 (4.4%)	2 (1.5%)	0 (0%)
Mesh exposure		2 (1.5%)	0 (0%)	0 (0%)

Data are mean ± SD (range); n(%). SD, Standard Deviation.

### 3.3 Anatomical outcomes, ultrasonic parameters, and subjective outcomes

Table 2 lists the objective outcome of the POP-Q measurements and the ultrasonic parameters. All POP-Q measurement points showed significant improvement at 3 months, 1 year, and 2 years postoperatively compared to preoperative values (*P* < 0.001). This confirms effective anatomical correction of the prolapse. Furthermore, reductions in Gh, elongation of Pb, and an increase in TVL indicate successful reconstruction of the genital hiatus and perineal body structures. Transperineal 3D ultrasound at 3 months postoperatively demonstrated significant decreases in URA, RVA, BND, and LHA compared to preoperative values (*P* < 0.05). These findings suggest improved pelvic floor dynamics related to urinary continence. A summary of the subjective outcomes of the PFDI-20 and PGI-I is listed in Table 2. PFDI-20 scores showed a continuous and significant decline at all postoperative follow-up points compared to the preoperative baseline (*P* < 0.001). This included marked decreases in the Pelvic Organ Prolapse Distress Inventory–6 score (reflecting prolapse symptoms, *p* < 0.001), the Colorectal Anal Distress Inventory–8 score (reflecting defecation symptoms, *p* < 0.001), and the Urinary Distress Inventory–6 score (reflecting urinary symptoms, *p* < 0.001). At the 2-year follow-up, PGI-I results indicated that 108 patients (80.0%) felt “very much improved” and 20 patients (14.8%) felt “much improved”, yielding an overall subjective satisfaction rate of 94.8%. During the follow-up period, POP recurred in 3 patients (2.2%) Table 3.

**Table 3:** Anatomical outcomes and subjective outcomes after biomechanical reconstruction of POP.

Parameters	Preoperative *n* = 135	3-month *n* = 135	1-year *n* = 135	2-year *n* = 135
POP-Q (cm)				
Aa^a^	2.2 ± 0.6	−2.6 ± 0.8	−2.4 ± 0.7	−2.3 ± 0.6
Ba^a^	2.8 ± 1.1	−2.4 ± 1.0	−2.2 ± 0.9	−2.1 ± 0.8
C^a^	0.5 ± 0.6	−6.4 ± 0.4	−6.2 ± 0.5	−6.0 ± 0.3
D^a^	−1.4 ± 0.9	−8.3 ± 0.7	−8.1 ± 0.8	−7.9 ± 0.6
Ap^a^	0.1 ± 0.6	−2.6 ± 0.3	−2.5 ± 0.3	−2.3 ± 0.4
Bp^a^	0.9 ± 0.6	−2.4 ± 0.4	−2.2 ± 0.3	−1.9 ± 0.2
Gh^a^	6.0 ± 0.5	3.4 ± 0.5	3.6 ± 0.3	3.7 ± 0.4
Pb^a^	2.0 ± 0.6	3.5 ± 0.5	3.2 ± 0.3	3.0 ± 0.4
TVL^a^	5.8 ± 0.9	8.4 ± 0.8	8.1 ± 1.0	7.9 ± 0.9
PFDI-20	151.2 ± 24.6^a^	54.1 ± 21.3^a^	17.7 ± 13.1^a^	10.1 ± 9.5^a^
Pelvic Organ Prolapse Distress Inventory–6	70.0 ± 11.3^a^	27.1 ± 11.9^a^	9.2 ± 7.1^a^	8.3 ± 6.3^a^
Colorectal Anal Distress Inventory–8	31.3 ± 10.9^a^	12.5 ± 7.1^a^	4.7 ± 4.6^a^	0.5 ± 1.7^a^
Urinary Distress Inventory–6	50.0 ± 13.9^a^	14.6 ± 8.5^a^	4.2 ± 3.9^a^	2.1 ± 3.3^a^
PGI-I				
Very much better		88 (65.2%)	99 (73.3%)	108 (80%)
Much better		42 (31.1%)	31 (23.0%)	20 (14.8%)
A little better		3 (2.2%)	3 (2.2%)	4 (3.0%)
No change		1(＜1%)	2 (1.5%)	3 (2.2%)
A little worse		0 (0%)	0 (0%)	0 (0%)
Much worse		0 (0%)	0 (0%)	0 (0%)
Very much worse		0 (0%)	0 (0%)	0 (0%)
URA^b^	81.5 ± 25.9	38.0 ± 13.7		
RVA^b^	164.7 ± 19.5	106.9 ± 18.9		
BND^b^	37.1 ± 13.3	12.7 ± 6.6		
HA^b^	30.7 ± 5.2	17.2 ± 4.8		

Data are expressed as mean ± SD; n(%). POP-Q, Pelvic Organ Prolapse Quantification System; PFDI-20, Pelvic Floor Distress Inventory-20; PGI-I, Patient Global Impression of Improvement; URA, Urethral Rotation Angle; RVA, Restrovesical Angle; BND, Bladder Neck Descent; LHA, Levator Hiatus Area (LHA).

a Preoperation vs. 3-month, preoperation vs. 1-year, and preoperation vs. 2-year (repeated-measures ANOVA, *P* < 0.001). .

b preoperative vs. 3-month. (Paired t-text, *p* < 0.05).

### 3.4 SUI outcomes

**Figure 3:**
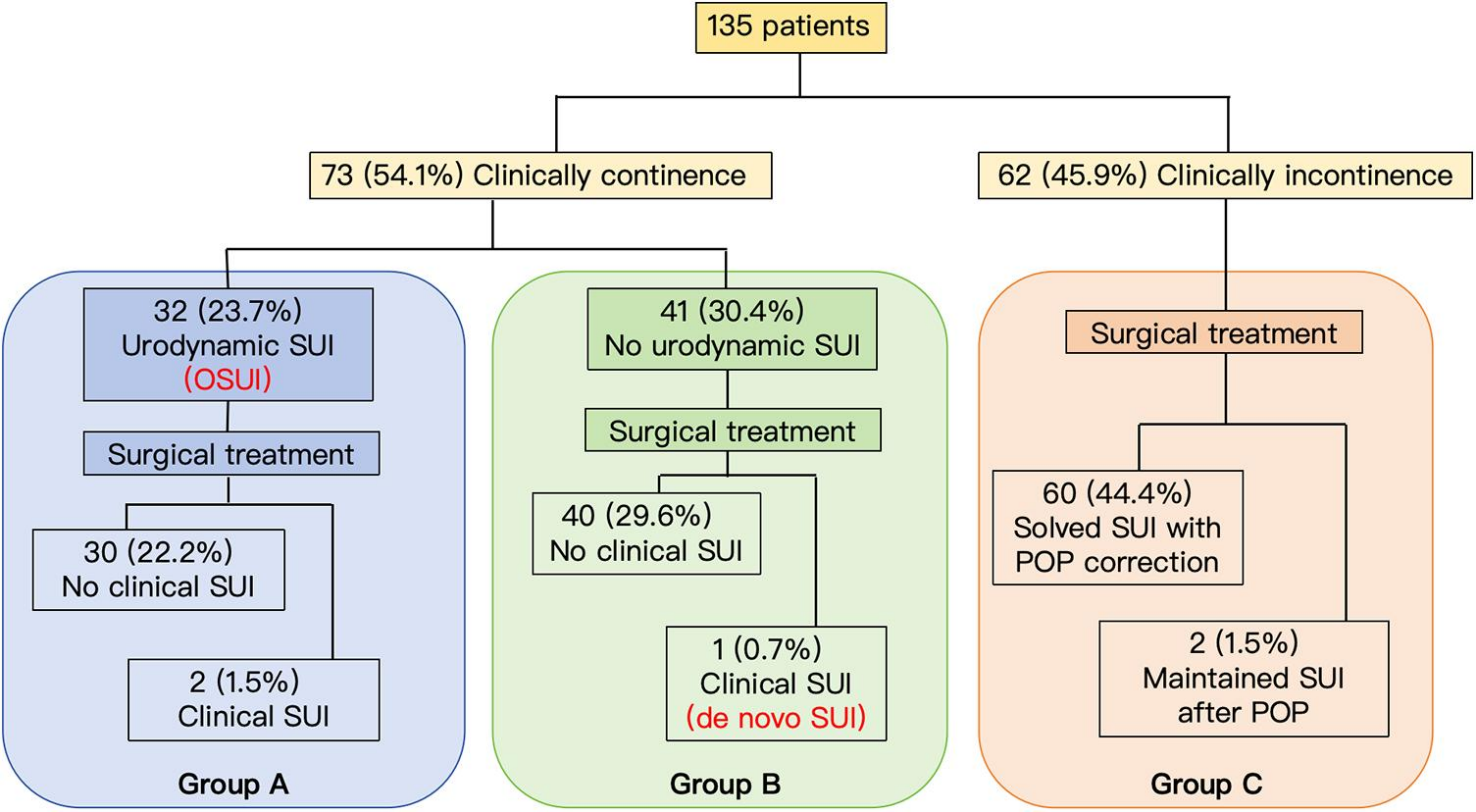
Outcomes of urinary incontinence. OSUI, Occult Stress Urinary Incontinence; SUI, Stress Urinary Incontinence; POP, Pelvic Organ Prolapse.

SUI outcomes at the 2-year follow-up were as follows: In Group A, only 2 patients (1.5%) still reported SUI symptoms postoperatively. In Group B, only 1 patient (0.7%) developed *de novo*SUI. In Group, only 2 patients (1.5%) continued to experience SUI symptoms after surgery. The overall incidence of SUI symptoms at 2 years was 3.7%. Figure 3 illustrates the SUI outcomes.

### 3.5 Follow-up complications

Two patients (1.5%) developed mesh exposure during follow-up. Both cases were classified as IUGA/ICS complication grade 2CT4S2. They healed well after local excision under local anesthesia. Five patients (3.7%) reported mild thigh or inguinal pain in the early postoperative period, with less than 1% requiring NSAIDs beyond 2 years postoperatively. At the 3-month follow-up, 7 patients (5.2%) reported overactive bladder symptoms and 6 patients (4.4%) reported urgency urinary incontinence. Most of these symptoms resolved with bladder training and/or solifenacin succinate treatment. The summary of follow-up Complications is shown in Table 2.

## 4 Discussion

In many cases, SUI only becomes apparent after prolapse reduction (occult SUI) or may emerge following prolapse surgery (*de novo*SUI). Accurately identifying which patients require a concomitant anti-incontinence procedure during POP repair remains a significant challenge in clinical practice. A large randomized controlled trial involving 313 POP patients without preoperative SUI symptoms reported an SUI incidence of 3.7% before prolapse reduction. However, this detection rate rose significantly to 6%–30% when different reduction methods were applied (21). This indicates that preoperative assessment alone is insufficient for accurately predicting postoperative continence risk, and a unified standard for judgment is currently lacking.

The optimal surgical strategy for patients with POP and concomitant SUI remains controversial. Costantini et al. (22, 23) randomized 47 patients with POP and SUI to compare sacrocolpopexy alone versus sacrocolpopexy combined with a Burch colposuspension. Their results showed that adding an anti-incontinence procedure did not improve postoperative SUI outcomes (22), a finding that persisted at the five-year follow-up (23). Another multicenter prospective RCT (24) compared performing MUS concurrently with POP surgery versus performing MUS three months later. The study found no significant difference in the one-year SUI cure rate between the two groups (RR 0.41). Notably, 29% of patients who did not receive initial MUS were cured of their SUI by the prolapse surgery alone. However, other evidence suggests that combined surgery can reduce postoperative SUI incidence. A randomized controlled trial involving 220 continent POP patients compared POP surgery alone to POP surgery combined with MUS. Among the 113 women who underwent POP surgery alone, 46 (40.7%) developed postoperative SUI, compared to 30 out of 107 women (28.0%) in the combined surgery group (RR 0.69) (5). Although combined surgery offers potential advantages for continence, pooled data from three randomized controlled trials (14–26) indicate that, compared to POP surgery alone, combined procedures are associated with a lower rate of postoperative urgency urinary incontinence but higher rates of voiding dysfunction and surgery-related adverse events. Therefore, from a clinical decision-making perspective, the ideal procedure should restore anatomical support while simultaneously reconstructing the physiological support structures relevant to urinary continence.

Our results demonstrate that pelvic floor biomechanical reconstruction achieved satisfactory anatomical restoration and symptomatic improvement in the short-to-mid term. All POP-Q points showed significant optimization, PFDI-20 scores decreased continuously, and PGI-I indicated high subjective satisfaction, with a POP recurrence rate of only 2.2%. More importantly, transperineal three-dimensional ultrasound at 3 months postoperatively showed marked improvement in parameters such as URA, RVA, BND, and LHA. This provides objective evidence that the procedure may improve urinary control by reconstructing the mechanical environment of the urethra and bladder neck. The overall incidence of incontinence symptoms at the two-year follow-up was 3.7%, a figure numerically lower than the ranges reported for some traditional procedures (5, 9). This highlights the potential unique advantage of this technique in reducing postoperative SUI risk. The key mechanisms are primarily reflected in the following two aspects:

1. Innovation in Anterior Compartment Repair and Restoration of Physiological Mechanics

In the anterior compartment repair, this technique involves placing the mesh flat from the mid-urethra to 2 cm anterior to the cervix. This design aligns better with physiological mechanical characteristics compared to traditional transvaginal mesh (TVM) implantation. From a functional mechanics perspective, the core of traditional TVM is to suspend and fix the cervix at the level of the sacrospinous ligament, which disregards the physiological mobility of the cervix. During increased intra-abdominal pressure (Valsalva maneuver), the cervical position should be below the level of the ischial spines. Such over-fixation disrupts the coordinated movement among pelvic organs, depriving the bladder, uterus, and rectum of their normal functional mobility. This inevitably increases the risk of complications such as SUI, recurrence of anterior/posterior vaginal wall prolapse, and constipation. By lowering the position of mesh coverage, this technique provides necessary support while preserving organ mobility. Furthermore, anterior mesh implantation, combined with folding sutures of the anterior vaginal wall during cystocele repair, can enhance bladder neck closure, increase urethral closure pressure, improve continence function, and potentially reduce the risk of mesh exposure.

2. Systematic Reconstruction of the Posterior Compartment Biomechanical Fulcrum

This technique establishes a stable stress fulcrum for the bladder neck by repairing the levator plate and significantly reducing the levator hiatus area. Our finite element study (26) confirmed that the mechanical fulcrum of the bladder neck is located at the junction of the levator plate and the external anal sphincter. By reinforcing this key area, the technique effectively limits excessive posterior-inferior displacement of the bladder neck during increased intra-abdominal pressure. Simultaneously, the reconstruction and elongation of the perineal body provide reliable direct support for the mid-to-distal urethra, significantly restricting excessive urethral displacement under stress, thereby better maintaining functional urethral length. This mechanism aligns with the findings of Yansheng X et al. (27), whose research also confirmed that reinforcing posterior pelvic support yields satisfactory continence outcomes. Notably, the unified conceptual model for pelvic floor morphology proposed by Professor DeLancey in 2024 also emphasizes the crucial role of the levator ani and perineal complex in maintaining pelvic floor function (28), which is consistent with the core concept of this technique.

Regarding safety, the incidence of perioperative and follow-up complications in this cohort was low and manageable. Intraoperative complications such as bladder perforation, and vaginal or rectal injuries were all managed successfully. Postoperative temporary urinary retention or inguinal pain mostly resolved within a short period. Mesh exposure rate was 1.5%, and all cases were successfully managed with local excision. Data from the International Federation of Gynecology and Obstetrics indicate that the mesh exposure rate for traditional TVM ranges from 4% to 19%, while sacrocolpopexy (SC) presents a comparatively lower rate (29). In our study, the anterior vaginal wall folding technique preserves tissue thickness and provides robust coverage, which may mitigate this risk. Symptoms of overactive bladder and urgency urinary incontinence in affected patients mostly improved with bladder training and medication.

The strengths of this study include a relatively large cohort size (*n* = 135) for a single-center procedure performed by a single surgeon, which minimizes operative bias. Additionally, the comprehensive dual-assessment approach, utilizing both objective anatomical/ultrasonographic measurements and validated subjective quality of life questionnaires (PFDI-20, PGI-I), strengthens the reliability of our findings. This study has several limitations. First, it is a single-center retrospective study, which carries inherent selection bias. Second, it lacks a direct comparison with traditional procedures such as TVM or sacrocolpopexy. Third, due to cost and the invasive nature of the test, postoperative urodynamic studies were not performed, preventing objective assessment of SUI outcomes. Fourth, other imaging modalities (such as pelvic MRI or MR defecography) were not used to further assess anatomical correlates. Fifth, 2 years is considered a short-to-mid-term follow-up; longer-term observation is needed to assess late-onset complications or POP recurrence. Finally, there is a lack of specific questionnaires (such as PISQ-12) to evaluate the impact of levatorplasty on sexual function. Future multicenter, prospective randomized controlled trials are needed to validate its long-term efficacy and further elucidate its biomechanical mechanisms.

In summary, pelvic floor biomechanical reconstruction achieves dual improvement in anatomy and function through physiological repair of the anterior compartment and systematic reconstruction of the posterior compartment fulcrum. It balances short-to-mid-term safety with patient subjective satisfaction. Its potential advantages require further confirmation through higher-quality research.

## 5 Conclusions

Pelvic floor biomechanical reconstruction can restore the normal anatomical position of pelvic organs while reconstructing urinary continence function, achieving synchronous improvement in structure and function. Short-to-mid-term follow-up shows low rates of postoperative SUI, mesh exposure, and recurrence, with overall manageable complications. Higher-quality evidence is still needed to verify its long-term efficacy and safety.

## Data Availability

The raw data supporting the conclusions of this article will be made available by the authors, without undue reservation.
